# Validation of glomerular filtration rate-estimating equations in Chinese children

**DOI:** 10.1371/journal.pone.0180565

**Published:** 2017-07-06

**Authors:** Ke Zheng, Mengchun Gong, Yan Qin, Hongmei Song, Ximin Shi, Yuan Wu, Fang Li, Xuemei Li

**Affiliations:** 1 Department of Nephrology, Peking Union Medical College Hospital, Chinese Academy of Medical Sciences, Beijing, China; 2 Department of Pediatrics, Peking Union Medical College Hospital, Chinese Academy of Medical Sciences, Beijing, China; 3 Department of Nuclear Medicine, Peking Union Medical College Hospital, Chinese Academy of Medical Sciences, Beijing, China; University of Sao Paulo Medical School, BRAZIL

## Abstract

**Background:**

Glomerular filtration rate (GFR) is essential for renal function evaluation and classification of chronic kidney disease (CKD), while the reference method in children are cumbersome. In the Chinese children, there was no data about GFR measured through plasma or renal clearance of the exogenous markers, and therefore no validated GFR-estimating tools in this population.

**Methods:**

We measured GFR with double-sample plasma clearance of ^99m^Tc-DTPA (mGFR) in 87 hospitalized children with renal injury. Using mGFR as the golden standard, we evaluate the efficiency of four different GFR estimation equations (the original and update Schwartz equation, the Filler equation, the CKiD equation) by statistical parameters of correlation, precision and accuracy.

**Results:**

In our population, mGFR was 97.0± 31.9 mL/min/1.73m^2^. The updated Schwartz equation, the Filler equation and the CKiD equation, produced eGFR with strong correlation with mGFR, strong explanation capacity of variance in mGFR, small bias, satisfactory performance in Bland-Altman analysis, high intra-class correlation coefficients, high ratio of eGFR within mGFR±10% and eGFR within mGFR±30%, good agreement in CKD staging between eGFR and mGFR. The original Schwartz equation produced eGFR with large bias, poor precision and accuracy.

**Conclusion:**

The validated equations to estimate GFR in our patients are the updated Schwartz equation, which is simple for bedside use, the Filler equation and the CKiD equation, which provide more accurate eGFR. The original Schwartz equation should not be applied to estimate GFR in Chinese children with kidney injuries.

## Introduction

Glomerular filtration rate (GFR) is the most commonly used indicator of kidney function. An accurate method of determining GFR is critical for prescribing optimal dosage of fluids and medications, monitoring for nephrotoxicity of antibiotics and chemotherapeutic agents, and assessing progression of renal disease[1]. However, determination of true GFR is time-consuming, costly, and difficult to perform for regular clinical use in children[2]. Thus, there is considerable interest in developing formulas to estimate GFR using endogenous markers such as creatinine or the other low molecular weight proteins, including Cystatin C[3] and beta-trace protein[4]. Estimated GFR is one of the key tools used in pediatric nephrology and it is demonstrated to be with non-inferiority, compared with measured GFR, in predicting CKD complications, including anemia, hyperphosphatemia, hyperkalemia, metabolic acidosis and progression to ESRD[5]. The original Schwartz (Schwartz1976) equation, devised for children in the mid-1970s, estimates GFR using serum creatinine (Scr), height, and an empirical constant[6]. This equation has been used since its development to direct clinical practice in pediatric nephrology. Recently, two new equations, the updated Schwartz (Schwartz2009) Equation and the CKiD equation, which were developed by CKiD research group, became the focus of research because of their significant improvement in the precision and accuracy of GFR estimation[7].

Although eGFR constitutes the fundamentals of pediatric nephrology, the tools to calculate that had never been developed or validated for the Chinese children, of whom the body shape, life style, and disease spectrum significantly differ from that of the children in Western countries. Previous data showed that application of MDRD equation, developed based on the American adult population, may produce significant bias when applied in Chinese adults[8]. The new CKD-EPI equation in adults, which combines sCr, sCysC and other demographic parameters, also needs modification with the ethnicity factors to be applied widely[9]. Thus, it is very important to evaluate the performance of a GFR-estimating equation in the local population before it is put into clinical practice.

The golden standard method of GFR measurement is the inulin urine clearance. However, it is time-consuming, difficult to perform, and most importantly, inaccessible in China. The reference method we used was the double plasma clearance of ^99m^Tc-DTPA, which had been proved to be accurate and recommended[10] as the alternative reference method to determine GFR. A previous study used the Gates method (scintigraphy) to measure GFR and compared several GFR-estimating equations in Chinese children with kidney injuries[11]. That study cannot provide convincing data to support the preferred use of any equation because the Gates method is affected by many factors other than GFR itself and can only be used to determine relative GFR[12].

Moreover, Cystatin C have been proven to be a more accurate marker for GFR estimation and CKD classification and risk stratification for adults and children[3]. Recent study proved that adding Cystatin C to the estimation system significantly improve the linearity and accuracy of the formula [13]. Yet no data existed about the use of Cystatin C for estimating the GFR in Chinese children. It’s necessary to evaluate the efficiency of this new biomarker, with which we can build up the equations that estimate GFRs more reliably in the Chinese children with kidney injuries.

Thus, the aim of this study was to evaluate the performance of commonly used equations biochemically based on serum creatinine (the original and update Schwartz equation), serum Cystatin C (the Filler equation) and their combination (the CKiD equation) in the Chinese children with kidney injuries.

## Methods

### Study patients

A total of 87 patients were enrolled in the study at the department of Nephrology or Pediatrics in Peking Union Medical College Hospital from September 2010 to May 2011. All of the patients were between 1 and 18 years old and had at least one of the following markers of renal injury: proteinuria or hematuria, elevated creatinine or Cystatin C, abnormal renal ultrasound or scintigraphy and abnormal pathologic findings. Patients were excluded if they were unable to provide informed consent, with acute kidney injury, severe edema, demonstrated systemic infection, massive ascites, a history of malignancy, recent surgery or being unable to corporate with scintigraphic study.

### Laboratory assessment

From September 2010 to May 2011, GFR was measured using plasma clearance of radio-labeled ^99m^Technetium-diethylene-triamine penta-acetic acid (^99m^Tc-DTPA) through a single bolus injection and plasma sampling at 2-hour and 4-hour after injection, as described elsewhere[10]. GFR was then corrected for standard body surface area(BSA) calculated using Haycock formula[14]. In the morning of the test day, height and weight was measured and blood was sampled for measurement of creatinine, urea and Cystatin C. Creatinine was measured through both Jaffe method and an isotope dilution-mass spectrometry (IDMS)-traceable enzymatic method. Cystatin C was measured through a particle-enhanced nephelometric assay. Urea was measured on an Olympus AU5421 Automatic Biochemical Analyzer.

### GFR estimation

From hundreds of GFR-estimating equations reported in literature, we selected four equations, classified according to the biochemical markers used in the formula, to perform validation because of the relatively large number of cases in the development and validation studies, approved method of GFR measurement, strict quality control in measuring the biochemical markers and out pilot study results. The formulae used to calculated eGFR are listed in Table 1.

**Table 1:** Details of the GFR estimating equations.

Equation	Formula	Measurement of GFR
Schwartz1976[6]	$55\;\times \;\frac{\text{H}}{\text{sCrJ}}\;\times \;1.273^{\text{boy}>13ys}$	Inulin
Schwartz2009[7]	$41.3\times \frac{\text{H}}{\text{sCrE}}$	Iohexol
Filler[15]	91.62 × (1/sCysC)^1.123^	^99m^Tc-DTPA
CKiD[7]	$39.1\;\times \;{\left[\frac{\text{H}}{\text{sCrE}}\right]}^{0.516}\times \;{\left[\frac{1.8}{\text{sCysC}}\right]}^{0.294}\times \;{\left[\frac{30}{\text{BUN}}\right]}^{0.169}\times \;\left[1.099^{\text{boy}}\right]\;\times \;{\left[\frac{\text{H}}{1.4}\right]}^{0.188}$	Iohexol

Units: Height (H,m), Body weight (W, kg), serum creatinine (sCr, mg/dL, sCrJ means creatinine measured by the Jaffe method and sCrE means creatinine measured by the IDMS-traceable enzymatic method), serum cystatin C (sCysC, mg/L), BUN (mg/dL)

### Statistical analysis

eGFR was calculated with the four equations as shown in Table 1. As recommended in the National Kidney Foundation Guidelines on CKD[16], Pearson correlation coefficient and linear regression analysis showed the explanatory capacity of eGFR for mGFR. The higher R-square means better equation performance in explaining the variance of GFR. Bias was calculated as Σ(*eGFR* − *nGFR*)/*N* and the performance is better if its 95% confidence interval (95%CI) includes zero or the p value in the paired t-test is larger than 0.05. 95% limits of agreement (95%LOA) was calculated as Bias±1.96×Standard Deviation_eGFR-mGFR_. 95% LOA reflects the range above and below mGFR that eGFR would fall in with a probability of 95%. Bland-Altman analysis and intraclass coefficient (ICC) were performed to compare the precision. The ratio of eGFR within mGFR±10% and mGFR±30% and the ratio of correct CKD staging by eGFR were used to compare the accuracy of GFR estimation of different equations.

### Ethics committee

The Independent Bioethical Committee of Scientific Researchers at the Peking Union Medical College Hospital/Chinese Academy of Medical Sciences approved this study. Written informed consents were obtained from the legal guardians of the children.

Authors had access to information that could identify individual participants after data collection

## Results

### Patient characteristics

The clinical characteristics of the study group (N = 87) are shown in Table 2. Forty-four patients are male (50.6%) and the median age is 12.0 years (IQR 10–15). All five stages of CKD are represented (CKD 1 59%, CKD2 26%, CKD 3–5 15%) and the major cause of CKD is glomerulonephritis (N = 55, 63.2%).

**Table 2:** Clinical characteristics of the study population.

Variables	Value
Male gender (%)	50.6
Diagnosis (%)	
Non LN-GN	33
LN	22
Non-GN	32
Age (years, median[IQR])	12.0 [10,15]
Height (m, median[IQR])	1.462 [1.343,1.572]
Body weight (kg, median[IQR])	42.0 [27.9, 52.8]
BSA*(m^2^, median[IQR])	1.310 [1.005, 1.501]
CrE (mg/dL, median[IQR])	0.536[0.425,0.764]
H(m)/CrE(mg/dL), median[IQR])	3.12 [2.24, 4.65]
CrJ (mg/dL, median[IQR])	0.622 [0.532, 0.814]
sCysC (mg/L, median[IQR])	0.86 [0.75,1.20]
BUN (mg/dL, median[IQR])	4.5 [3.6,5.8]
mGFR (ml/min *per* 1.73m^2^, mean±SD)	97.0± 31.9

* calculated as described in Methods;

IQR, interquatile range; LN, lupus nephritis; GN, glomerulonephritis; BSA, body surface area; BMI, body mass index; CrE, enzymatic serum creatinine traceable to IDMS;CrJ, Jaffe-method serum creatitine, sCysC, serum cystatin C; mGFR, normalized measured GFR.

### Equation performance

The average eGFRs calculated by the four different equations and the results of correlation and precision analysis are shown in Table 3 while the accuracy analysis results in Table 4.

**Table 3:** Correlation and precision of GFR estimation by different equations.

Equation	eGFR mL/min×1.73m^2^	Bias	r_*Pearson*_	*R*^2^	95%LOA	ICC	Slope
Schwartz1976	127.1±42.1	30.1*	0.61	0.58	-37.0, 97.3	0.61	0.35^φ^
Schwartz2009	106.9±37.5	9.8*	0.69	0.63	-44.8, 64.4	0.79	0.20^φ^
Filler	100.0±38.5	3.3	0.70	0.67	-51.6, 58.3	0.81	0.27^φ^
CKiD	110.4±34.6	13.4*	0.75	0.72	-32.9, 59.6	0.82	0.10

LOA, limits of agreement;

$Bias=\sum_{i=1}^{87}\left(eGFR_{i}-mGFR_{i}\right)/87$

* designating a p value <0.01 in the paired t-test;

95% LOA = Bias±1.96×SD of (eGFR-mGFR); ICC, intra-class correlation coefficient, showing the degree of absolute agreement among measurements based on estimating the reliability of averages of *k* ratings. Slope, the slope of regression line in the Bland-Altman Analysis;

^φ^ designating the p value<0.05

**Table 4:** Accuracy of GFR estimation by different equations.

Equation	% of eGFR within mGFR±10%	% of eGFR within mGFR±30%	% of correct CKD staging
Schwartz1976	15	40	71
Schwart2009	24	63	70
Filler	37	74	71
CKiD	27	67	76

In correlation analysis, the eGFRs produced by all the four equations have significant linear correlation with mGFR (p<0.0001), and the linear regression demonstrated strong explanatory capacity of eGFR produced by Schwartz2009 (*R*^*2*^ = 0.63), Filler (*R*^*2*^ = 0.67) and CKiD (*R*^*2*^ = 0.72).

In the precision analysis, the Filler equation is the only one that produced eGFR without a significant bias from mGFR in the paired t-test (bias 3.3 mL/min per 1.73m^2^, 95% CI [-2.6, 9.3] mL/min per 1.73m^2^). The original and updated Schwartz equation and the CKiD equation significantly over-estimate GFRs, with the biases of 30.1 (95% CI [22.8, 37.4]), 9.8 (95% CI [3.8, 15.7]) and 13.4 (95% CI [8.4, 18.4]) mL/min per 1.73m^2^, respectively.

**Fig 1:**
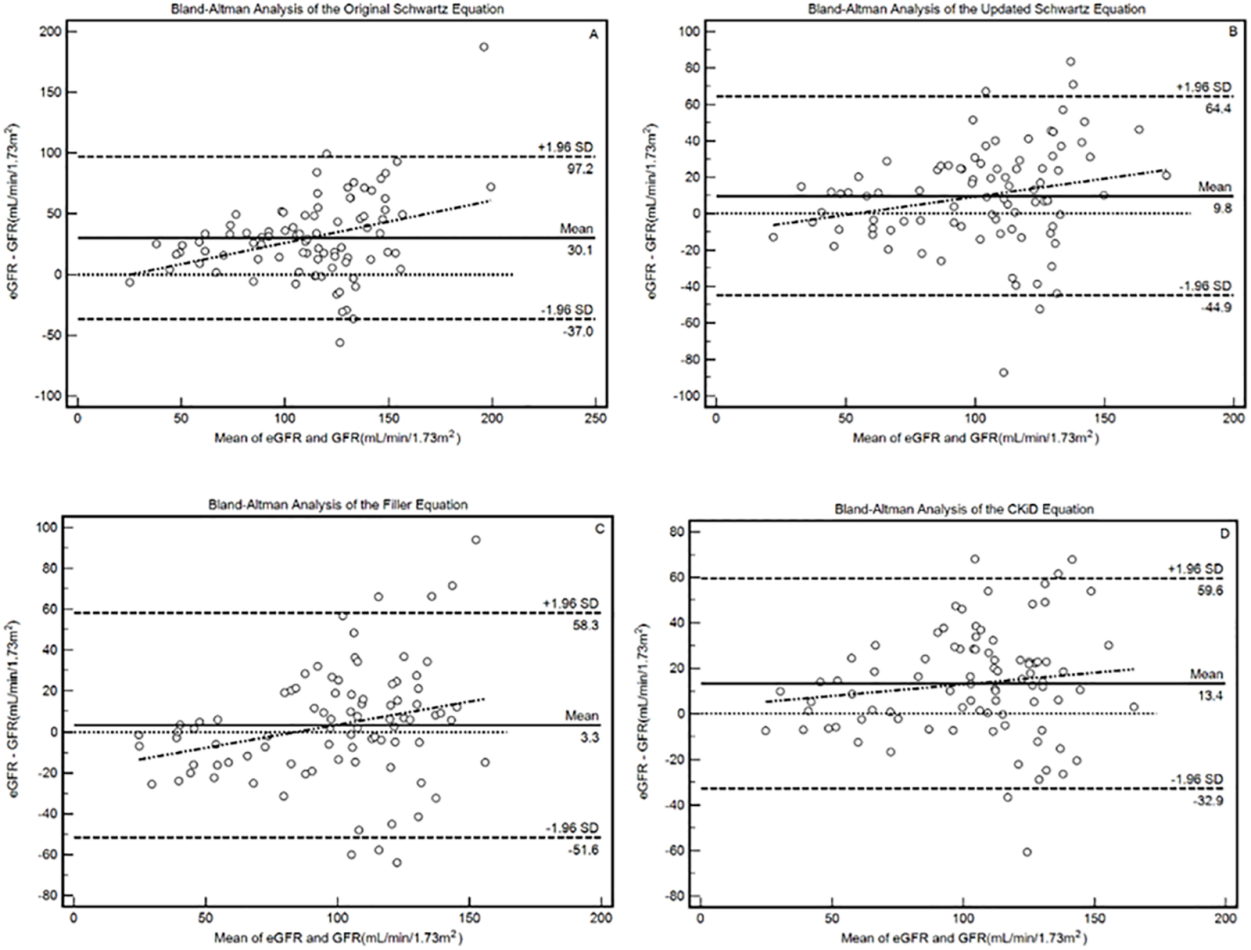
The Bland-Altman analysis of the four equations. The slopes in the Bland-Altman analysis of Schwartz1976 (original Schwartz, A), Schwartz2009 (updated Schwartz, B), Filler (C) and CKiD (D) equations are 0.35 (95% CI [0.14, 0.56]), 0.20 (95% CI [0.02, 0.38]), 0.23 (95% CI [0.05, 0.23]) and 0.10, (95% CI [-0.06, 0.26]), respectively.

Agreement between eGFR and mGFR, measured by intraclass correlation coefficient (ICC) was calculated and highest ICCs are produced by Filler (0.81) and CKiD (0.82). The 95% CI of ICC for Schwartz1976 (95% CI [0.06, 0.81]) approached zero, indicating poor agreement between eGFR and mGFR. The Bland-Altman analysis results are shown in Fig 1. The narrowest width of 95% LOA are produced by the CKiD equation. The significant slope of the regression line in Bland-Altman analysis indicates the increased bias with increase GFR level. The CKiD equation produced a regression line slope without significance (Slope = 0.10, 95% CI [-0.06, 0.26]), indicating its stable performance throughout the different stages of kidney function impairment. The slopes in the Bland-Altman analysis of Schwartz1976, Schwartz2009 and Filler equations are 0.35 (95% CI [0.14, 0.56]), 0.20 (95% CI [0.02, 0.38]) and 0.23 (95% CI [0.05, 0.23]).

In the accuracy analysis, the ratio of eGFR within mGFR±10% and mGFR±30% were calculated. The highest ratio of eGFR within mGFR±10% and within mGFR±30% were produced by Filler (37% and 74%, respectively). The highest ratio of correct staging were produced by CKiD (76%).

## Discussion

This is the first study validating the GFR estimation equations in the Chinese children with kidney injuries, using the reference GFR measured by double-sample ^99m^Tc-DTPA plasma clearance, which is widely accepted as the substitute of inulin renal clearance for accurate evaluation of GFR.

Among the three serum creatinine-based equations, the original Schwartz (Schwartz1976) equation significantly overestimated renal function in our study, with biases of 30.3 mL/min per 1.73m^2^ (p<0.05). Poor correlation (Pearson correlation coefficient 0.61), weak explanation capacity (R^2^<0.6 in the linear regression) and unsatisfactory accuracy (with only 40% eGFR within mGFR±30%) were also observed, indicating a necessary modification of this equation, similar to that for the MDRD equation in adults [8]. A European study [13] including 238 CKD children proved an even larger bias of the original Schwartz equation (bias 50 mL/min per 1.73m^2^). The original Schwartz equation was developed using an end-point Jaffe reaction, which is less specific than the enzymatic method used in the Schwartz2009 and CKiD equation. The need for a local specific correction of the *κ* has been well demonstrated[17], which has never been done here in China because there had been no measured GFR to be taken as reference. The original Schwartz equation was not validated to be used in Chinese CKD children.

The updated Schwartz (Schwartz2009) equation, based on IDMS-traceable enzymatic creatinine and height and designed for bedside use because of its simple structure, showed acceptable, though significant, bias (10.1 mL/min per 1.73m^2^, P<0.05), satisfactory accuracy (with 75% eGFR within mGFR±30%, 71% of correct CKD staging based on eGFR). The overestimation is concordant with studies in other countries[18]. The original population was a cohort of 349 North American children with mild to severe CKD (median GFR 41mL/min per 1.73m^2^) and notable growth retardation[7]. External validation of this formula in children with greater GFR and no significant growth retardation is necessary. There are evidence proving that age may play an important role in determining the GFR estimation of the updated Schwartz equation [19]. At present, the updated Schwartz equation is recommended as a useful and reliable tool for the Chinese pediatrician and nephrologists to evaluate renal function of the children, because of the simple structure of the formula, the generalized application of the enzymatic Creatinine measurement, and more importantly, lack of a formula developed according to the local dataset. However, measurement with exogenous markers, such as ^99m^Tc-DTPA or Iohexol, may be performed if an accurate GFR has to be determined.

Cystatin C has been regarded as a promising marker of renal function evaluation because of its stable production rate since 12 month after birth, independence of muscle mass, negligible clearance through non-renal pathways[20]. Several studies proved its improved performance in GFR estimation [13]. Height and body weight, which change with age significantly in children and therefore affect creatinine level, do not have a clinically relevant effect on cystatin C GFR in children[21]. Filler equation, based solely on Cystatin C, showed excellent performance, though its structure is simple. The eGFR produced by Filler equation showed strong correlation with mGFR (r = 0.69), good precision (bias 3.5 mL/min per 1.73m^2^, p = 0.2) and satisfactory accuracy (with 35% eGFR within mGFR±10% and 76% eGFR within mGFR±30%, 69% of correct CKD staging based on eGFR). Recent evidence showed that Cystatin C-based equations produced a more accurate CKD classification in adults, which lead to further better risk stratification in adults. Here we presented the first study to prove the use of Cystatin C, a widely measured biochemical marker in China, in GFR estimation of Chinese children and the selected equation is the Filler equation, which doesn’t contain the age parameter. We proved that CysC-based prediction equations are at least as good as creatinine-based formulas, as reported in systemic review[22].

The CKiD equation, combing enzymatic creatinine and Cystatin C, does not showed significantly better performance than the other three equations as expected and the ratio of eGFR within mGFR±30% and correct CKD staging were not as high as reported in original literature or other validating studies[23]. There are several possible explanations for this. Firstly, the methods to measure GFR were different in the two studies. In our study, we used ^99m^Tc-DTPA transformed double-compartment plasma clearance as measured GFR, while the CKiD study used iohexol measured double-compartment plasma clearance[24], which requiring more samplings (3 vs. 5) and longer procedure time (4 hours vs. 6 hours). Secondly, the GFR levels and primary kidney diseases differed a lot between the two groups. Mean mGFR are significantly different (97.0 vs. 41.3 ml/min per 1.73m^2^, p<0.0001). The proportion of patients with the diagnosis of non-glomerulonephritis in our study group is significantly lower than that in the CKiD research (13% vs. 63%, p<0.0001). Moreover, the precision of all the four equations are not satisfactory, according to the Bland-Altman Analysis (shown in Fig 1). This is in concordance with the published studies[25]. Currently, we have to accept a precision (as defined in the Bland-Altman plot) of ±20% in adults and ±30–40% in children. Increase in sample size, standardization of the measurement of biochemical markers, extended enrollment of all CKD stages, especially the severely damaged cases, are important ways to improve the precision of the formulas. The slope of its Bland-Altman plotting regression line is a marker to evaluate the stability of GFR estimation of equations across different levels of true GFR. The CKiD equation is the only one of which the slope is 0.10 and its 95% CI covered zero. So the CKiD equation is stable across this study population, mainly CKD stage 1 to 3. This result can complement the original study since the developing data and internal validation data included mainly the children with moderately-to-severely damaged renal function [7]. Thus, we recommend the CKiD equation for the GFR estimation in children with mildly damaged or normal renal function.

We concluded that the validated GFR-estimating equations for Chinese children with CKD were the updated Schwartz equation, Filler equation, and CKiD equation. The CKiD equation is recommended for the GFR estimation in children with moderately damaged or normal renal function, while the updated Schwartz equation is recommended for simple bedside use. Application of serum Cystatin C in GFR estimation can improve the renal function evaluation. The original Schwartz equation, based on Jaffe-method creatinine, should not be applied to estimate GFR in Chinese children with kidney injuries.

There are several limitations of our study. Firstly, only three blood samples (at 0h, 2h and 4h) were obtained and the data were insufficient to plot the double-compartment clearance curve. We have to use transforming equations to get the double-compartment clearance. Secondly, we need to enroll more children with more severe renal function impairment to enhance the validity of our conclusion for those at higher stages of CKD. Thirdly, the clinical background in this hospital, which is a nation-wide referral center for renal biopsy, made it difficult to enroll patients group with a more extended disease spectrum. Collaboration with other pediatric centers in Beijing would help us to solve this problem in the future study.

## Supporting information

S1 Table Children GFR database.xlsx. The detailed information of this study.
(XLSX)

## References

[1] Hogg RJ, Furth S, Lemley KV, Portman R, Schwartz GJ, Coresh J, et al National Kidney Foundation's Kidney Disease Outcomes Quality Initiative clinical practice guidelines for chronic kidney disease in children and adolescents: evaluation, classification, and stratification. Pediatrics. 2003;111(6 Pt 1):1416–21. Epub 2003/06/05. . 12777562 10.1542/peds.111.6.1416

[2] Schwartz GJ, Work DF. Measurement and estimation of GFR in children and adolescents. Clin J Am Soc Nephrol. 2009;4(11):1832–43. Epub 2009/10/13. doi: 10.2215/CJN.01640309 . 19820136 10.2215/CJN.01640309

[3] Ylinen EA, Ala-Houhala M, Harmoinen AP, Knip M. Cystatin C as a marker for glomerular filtration rate in pediatric patients. Pediatric nephrology. 1999;13(6):506–9. Epub 1999/08/19. doi: 10.1007/s004670050647 . 10452279 10.1007/s004670050647

[4] White CA, Akbari A, Doucette S, Fergusson D, Hussain N, Dinh L, et al Estimating GFR using serum beta trace protein: accuracy and validation in kidney transplant and pediatric populations. Kidney Int. 2009;76(7):784–91. Epub 2009/07/25. doi: 10.1038/ki.2009.262 . 19625992 10.1038/ki.2009.262

[5] Hsu CY, Propert K, Xie D, Hamm L, He J, Miller E, et al Measured GFR Does Not Outperform Estimated GFR in Predicting CKD-related Complications. J Am Soc Nephrol. 2011;22(10):1931–7. Epub 2011/10/04. doi: 10.1681/ASN.2010101077 . 21921144 10.1681/ASN.2010101077 PMC3187187

[6] Schwartz GJ, Haycock GB, Edelmann CM Jr., Spitzer A. A simple estimate of glomerular filtration rate in children derived from body length and plasma creatinine. Pediatrics. 1976;58(2):259–63. Epub 1976/08/01. . 951142

[7] Schwartz GJ, Munoz A, Schneider MF, Mak RH, Kaskel F, Warady BA, et al New equations to estimate GFR in children with CKD. J Am Soc Nephrol. 2009;20(3):629–37. Epub 2009/01/23. doi: 10.1681/ASN.2008030287 ; 19158356 10.1681/ASN.2008030287 PMC2653687

[8] Ma YC, Zuo L, Chen JH, Luo Q, Yu XQ, Li Y, et al Modified glomerular filtration rate estimating equation for Chinese patients with chronic kidney disease. J Am Soc Nephrol. 2006;17(10):2937–44. Epub 2006/09/22. doi: 10.1681/ASN.2006040368 . 16988059 10.1681/ASN.2006040368

[9] Stevens LA, Claybon MA, Schmid CH, Chen J, Horio M, Imai E, et al Evaluation of the Chronic Kidney Disease Epidemiology Collaboration equation for estimating the glomerular filtration rate in multiple ethnicities. Kidney Int. 2011;79(5):555–62. Epub 2010/11/26. doi: 10.1038/ki.2010.462 . 21107446 10.1038/ki.2010.462 PMC4220293

[10] Piepsz A, Ham R, De Sadeleer C. Guidelines for the measurement of glomerular filtration rate using plasma sampling. Nucl Med Commun. 2005;26(2):175–6; author reply 6–8. Epub 2005/01/20. . 15657513 10.1097/00006231-200502000-00016

[11] Wang F, Yao Y, Zhu SN, Huang JP, Xiao HJ, Ding J, et al [Evaluation of the applicability of three prediction equations for estimating glomerular filtration rate in children with chronic kidney disease]. Zhonghua Er Ke Za Zhi. 2010;48(11):855–9. Epub 2011/01/11. . 21215031

[12] Piepsz A, Blaufox MD, Gordon I, Granerus G, Majd M, O'Reilly P, et al Consensus on renal cortical scintigraphy in children with urinary tract infection. Scientific Committee of Radionuclides in Nephrourology. Semin Nucl Med. 1999;29(2):160–74. Epub 1999/05/13. . 10321827 10.1016/s0001-2998(99)80006-3

[13] Chehade H, Cachat F, Jannot AS, Meyrat BJ, Mosig D, Bardy D, et al New combined serum creatinine and cystatin C quadratic formula for GFR assessment in children. Clin J Am Soc Nephrol. 2014;9(1):54–63. doi: 10.2215/CJN.00940113 ; 24202134 10.2215/CJN.00940113 PMC3878689

[14] Haycock GB, Schwartz GJ, Wisotsky DH. Geometric method for measuring body surface area: a height-weight formula validated in infants, children, and adults. J Pediatr. 1978;93(1):62–6. Epub 1978/07/01. . 650346 10.1016/s0022-3476(78)80601-5

[15] Filler G, Foster J, Acker A, Lepage N, Akbari A, Ehrich JH. The Cockcroft-Gault formula should not be used in children. Kidney Int. 2005;67(6):2321–4. Epub 2005/05/11. doi: 10.1111/j.1523-1755.2005.00336.x . 15882274 10.1111/j.1523-1755.2005.00336.x

[16] Hogg RJ, Furth S, Lemley KV, Portman R, Schwartz GJ, Coresh J, et al National Kidney Foundation's Kidney Disease Outcomes Quality Initiative clinical practice guidelines for chronic kidney disease in children and adolescents: evaluation, classification, and stratification. Pediatrics. 2003;111(6 Pt 1):1416–21. . 12777562 10.1542/peds.111.6.1416

[17] Dubourg L C P, Baverel G, Hadj-Aïssa A. Schwartz formula has to be adapted to the method of creatinine determination. Pediatric nephrology. 2006;21:1526.

[18] Uemura O, Nagai T, Ishikura K, Ito S, Hataya H, Gotoh Y, et al Creatinine-based equation to estimate the glomerular filtration rate in Japanese children and adolescents with chronic kidney disease. Clinical and experimental nephrology. 2013 doi: 10.1007/s10157-013-0856-y . 24013764 10.1007/s10157-013-0856-y

[19] De Souza VC, Rabilloud M, Cochat P, Selistre L, Hadj-Aissa A, Kassai B, et al Schwartz formula: is one k-coefficient adequate for all children? PloS one. 2012;7(12):e53439 Epub 2013/01/04. doi: 10.1371/journal.pone.0053439 ; 23285295 10.1371/journal.pone.0053439 PMC3532344

[20] Bokenkamp A, Domanetzki M, Zinck R, Schumann G, Byrd D, Brodehl J. Cystatin C—a new marker of glomerular filtration rate in children independent of age and height. Pediatrics. 1998;101(5):875–81. Epub 1998/05/23. . 9565418 10.1542/peds.101.5.875

[21] Sharma AP, Kathiravelu A, Nadarajah R, Yasin A, Filler G. Body mass does not have a clinically relevant effect on cystatin C eGFR in children. Nephrology, dialysis, transplantation: official publication of the European Dialysis and Transplant Association—European Renal Association. 2009;24(2):470–4. doi: 10.1093/ndt/gfn505 . 18786974 10.1093/ndt/gfn505

[22] Andersen TB, Eskild-Jensen A, Frokiaer J, Brochner-Mortensen J. Measuring glomerular filtration rate in children; can cystatin C replace established methods? A review. Pediatric nephrology. 2009;24(5):929–41. Epub 2008/10/08. doi: 10.1007/s00467-008-0991-y . 18839216 10.1007/s00467-008-0991-y

[23] Andersen TB, Jodal L, Erlandsen EJ, Morsing A, Frokiaer J, Brochner-Mortensen J. Detecting reduced renal function in children: comparison of GFR-models and serum markers. Pediatric nephrology. 2013;28(1):83–92. doi: 10.1007/s00467-012-2268-8 . 22945867 10.1007/s00467-012-2268-8

[24] Schwartz GJ, Furth S, Cole SR, Warady B, Munoz A. Glomerular filtration rate via plasma iohexol disappearance: pilot study for chronic kidney disease in children. Kidney Int. 2006;69(11):2070–7. Epub 2006/04/14. doi: 10.1038/sj.ki.5000385 . 16612328 10.1038/sj.ki.5000385

[25] Gretz N, Schock D, Sadick M, Pill J. Bias and precision of estimated glomerular filtration rate in children. Pediatric nephrology. 2007;22(2):167–9. doi: 10.1007/s00467-006-0379-9 . 17123113 10.1007/s00467-006-0379-9

